# Prevalence and investigation of Cytomegalovirus (HCMV) in blood donors from the main blood establishment in Rio de Janeiro/Brazil

**DOI:** 10.1016/j.bjid.2025.104508

**Published:** 2025-02-07

**Authors:** Agildo da Silva Oliveira, Jéssica Gonçalves Pereira, Gabrielle Tantos Nunes, Ivanildo Pedro de Sousa Junior, Dmitry José de Santana Sarmento, Josiane Iole França Lopes, Luiz Amorim Filho, Vanessa Salete de Paula

**Affiliations:** a Instituto Estadual de Hematologia Arthur de Siqueira Cavalcanti/HEMORIO, Rio de Janeiro, RJ, Brazil; b Instituto Oswaldo Cruz (Fiocruz), Laboratório de Virologia Molecular e Parasitologia, Rio de Janeiro, RJ, Brazil; c Universidade Estadual da Paraíba, Faculdade de Odontologia, Departamento de Diagnóstico Oral, Araruna, PB, Brazil; d Centro Universitário Facisa (UNIFACISA), Departamento de Medicina, Campina Grande, PB Brazil

**Keywords:** Human Cytomegalovirus (HCMV), Blood donation, Blood transfusion

## Abstract

**Background:**

Human Cytomegalovirus (HCMV) remains a significant cause of morbidity and mortality among pregnant women and immunocompromised patients. HCMV transmission can occur through blood transfusions and typically results in asymptomatic infections in newborns and young individuals or causes symptoms like infectious mononucleosis when symptomatic infections arise. HCMV infection poses a notable risk to transfusion recipients, particularly in vulnerable groups such as premature newborns and immunosuppressed patients. The risk persists even after prophylaxis ends, especially in patients who undergo organ transplantation and receive blood or blood products from a seropositive donor while being seronegative themselves (D+/R-).

**Materials and methods:**

Here, we investigated the serological and molecular prevalence of HCMV among 980 blood donors from the main blood bank in Rio de Janeiro, Brazil, using chemiluminescence and real-time PCR (TaqMan). The data underwent univariate, bivariate, and multivariate statistical analyses using the SPSS program, version 20.0.

**Results:**

The average age of donors was 38.53 years, with a majority being male (53.9 %). The prevalence of cytomegalovirus was 88.5 %, and HCMV DNA was detected in 1.2 % of the samples.

**Discussion:**

Given that there are approximately 100,000 blood donations per year, this prevalence rate is considerably high compared to that in developed countries. These findings underscore the critical need for ongoing surveillance and molecular testing to ensure the safety of blood supplies.

## Introduction

Human Cytomegalovirus (HCMV), a member of the Orthoherpesviridae family, falls under the subfamily Betaherpesvirinae and the genus Cytomegalovirus.^1^ It possesses a double-stranded linear DNA genome ranging from 230‒240 kb, encased in a protein matrix known as the tegument. This matrix is further enveloped by a lipid bilayer embedded with viral glycoproteins.^2^^,^^3^ Recognized as the largest human herpesvirus, HCMV is particularly adept at causing significant damage to the host's immune system, posing a serious threat to immunosuppressed individuals.^4^, ^5^, ^6^ In immunocompetent individuals, primary HCMV infection is generally asymptomatic. However, in certain cases, it can progress to a condition resembling infectious mononucleosis, characterized by symptoms such as fever, pain, lymphadenopathy, and hepatomegaly.^7^ The symptoms can vary depending on age, health status, and the route of transmission and may extend to neurological complications, impairing hearing, and vision. Other associated symptoms and morbidities include premature birth, jaundice, microcephaly, seizures, and skin rash.^8^^,^^9^ Like other herpesviruses, HCMV can enter a latent state. Following initial infection, it remains dormant within the host.^8^ The genetic material of HCMV is detectable in various cell types, including monocytes, macrophages, neutrophils, lymphocytes, and endothelial cells. Reactivation of the virus can occur under immunosuppressive conditions, leading to viral replication and the subsequent onset of clinical symptoms.^10^ The virus's ability to maintain latency and reactivate is linked to its numerous immune evasion strategies.

In individuals who have undergone transplantation, HCMV infection can lead to both direct and indirect effects. Direct effects include the development of clinical symptoms, while indirect effects arise from the immune response and antigenic stimulus triggered by the virus, which can increase the risk of organ rejection and dysfunction.^11^^,^^12^ Specific immunity to HCMV is crucial in determining the severity and outcome of the infection. It is well-documented that immunocompromised individuals, those who are immunosuppressed or neonates, face a higher risk of developing severe diseases caused by HCMV.^8^^,^^11^^,^^13^ HCMV can be transmitted through various routes, including saliva, urine, blood, and breast milk, with sexual transmission through sperm also being significant. Additionally, Transfusion-Transmitted HCMV (TT-HCMV) poses a serious risk of morbidity and mortality for immunocompromised patients.^8^ Therefore, it can be concluded that HCMV infection may occur vertically, horizontally, and congenitally.^2^ The potential for severe health implications makes the transmission of HCMV through blood transfusions particularly concerning for several groups, including immunocompromised patients, newborns, other vulnerable individuals, and transplant recipients.^14^

The efficacy of TT-HCMV from seropositive donors ranges from 0 % to 12 %, largely depending on the recipient's degree of immunosuppression and anti-CMV status.^15^ When congenital transmission occurs, the rate of Central Nervous System (CNS) involvement is between 5 % and 10 % in affected newborns.^16^ This condition can manifest as encephalitis, seizures, and microcephaly and may lead to further complications such as deafness and delayed psychomotor development.^3^

Studies indicate that a significant proportion of patients requiring a blood transfusion belong to immunosuppressed, premature, and newborn groups. These individuals either have compromised or immature immune systems or are particularly susceptible to HCMV. Although serological testing for HCMV in blood donors reduces the risk of transmission through transfused blood and blood products,^4^^,^^17^ the virus's capacity for latency and reactivation means that patients who are IgG positive for HCMV can still have circulating HCMV DNA.^6^^,^^18^

Currently, there are no universally accepted international guidelines specifically dedicated to preventing TT-CMV. The management of TT-CMV largely depends on regional or national guidelines and practices, which can vary significantly. Although cell-free viral particles do exist and circulate during primary infection or viral reactivation, they are not considered a major mechanism for transfusion transmission. This is particularly true when transfused cellular blood components undergo leukoreduction filtration, a common processing method. After leukoreduction, the primary remaining risk of TT-CMV is linked to cell-free viruses present in the plasma of blood donors following primary infection or reactivation. Despite advancements in blood component processing, cell-free CMV continues to be a concern as it can persist through all processing stages.^4^

In this context, studies on the prevalence and detection of HCMV are crucial to understanding the current situation and ensuring the safety of recipients receiving blood products in hospitals and blood transfusion services, especially those who are immunosuppressed, immunocompromised, or have undergone transplantation. Therefore, this study provides important data on the necessity of HCMV testing in blood donors and assesses the risk of infection for recipients.

## Materials and methods

### Study design and population

A cross-sectional study was conducted at the Arthur de Siqueira Cavalcanti State Institute of Hematology (HEMORIO) in Rio de Janeiro, Brazil, enrolling 980 blood donors who visited the blood center in 2021. To determine the necessary sample size (N), the maximum prevalence of HCMV in Rio de Janeiro was estimated.^19^ The calculation was based on an annual sampling of 102,060; with a sampling error of 5 % and a confidence interval of 95 %, yielding a minimum required sample size of 322 blood donors. Demographic and behavioral data were collected through a questionnaire. Inclusion criteria required participants to be 18 years of age or older and to have signed a free and informed consent form for blood donation. Donors with inconsistent or unavailable registration data or whose samples showed hemolysis or lipemia were excluded. This study was approved by the Human Research Ethics Committee at the Arthur Siqueira Cavalcanti State Institute of Hematology ‒ HEMORIO, under approval n° 4.924.518, CAAE: 49342821.6.1001.5267, in accordance with resolution 466/12.

### Sample collection

Samples were collected at HEMORIO via peripheral venipuncture into PPT tubes containing K2 EDTA anticoagulant and polyester gel, which aids in separating the plasma and cellular fractions from whole blood. A 2 mL aliquot of each sample was transported to the NAT HEMORIO laboratory, where plasma and blood cell fractions were separated by centrifugation at 4000 rotations per minute (rpm) for 10min. The samples were then stored in a freezer at −80 °C for subsequent serological and molecular analysis.

### Chemiluminescence serology

#### Anti-HCMV IgG and anti-HCMV IgM

Anti-HCMV IgG and anti-HCMV IgM levels were measured using chemiluminescence (CLIA) on the DiaSorin - LIAISON® XL equipment. The results for anti-HCMV IgG concentrations, expressed in U/mL, range from 5.0 to 180 U/mL and are read automatically. Concentrations below 12.0 U/mL are interpreted as negative, those between 12.0 and 14.0 U/mL are considered indeterminate, and levels equal to or above 14.0 U/mL are classified as positive. Similarly, anti-HCMV IgM concentrations are also expressed in U/mL. The measurement range for IgM spans from 5.0 to 140 U/mL. Values below 18.0 U/mL are interpreted as negative, those between 18.0 and 22.0 U/mL are considered indeterminate, and concentrations equal to or above 22.0 U/mL are deemed positive.

### Extraction of viral DNA

#### Pool preparation

The detection of DNA was conducted in minipools of six samples, each containing 100 µL of serum, using the JANUS® automatic platform (Perkin Elmer®). After pooling, the samples were transferred to secondary 15 mL polystyrene tubes. If a pool tested positive, all six samples within it were then tested individually. The tests utilized a calibrator particle to validate the reactions, both for pooled and individual samples. The extraction of viral DNA was automated using the BioRobotMDx (Qiagen) equipment, along with the NAT HIV/HCV/HBV extraction kit from Bio-Manguinhos. This extraction method relies on the selective binding properties of the silica membrane. The eluted product is devoid of serum albumin, other proteins, nucleases, and inorganic and organic salts, thereby eliminating potential interferences.

### Detection of viral DNA

After nucleic acid extraction, the samples underwent real-time PCR (qPCR) analysis using the QuantStudio 3 Real-Time PCR Systems (Thermo Fisher Scientific) with the High Pure Viral Nucleic Acid kit (Roche Applied Science, Germany), following the manufacturer's instructions. For the detection of HCMV, the sequence probe CCGTATTGGTGCGCGATTCTGTTCAA-NFQ-MGB was used, along with oligonucleotide primers: sense 5′GGCCGTTACTGTCTGCAGGA3′ and antisense 5′GGCCTGGTAGTGAAAATTAATGGT3′ targeting the conserved region UL54, as per the established protocol.^20^

### Data analysis and statistics

Data were tabulated in Microsoft Excel and transferred for analysis to SPSS, version 20.0 (SPSS, Inc., Chicago, IL, USA). The normality test, using the Shapiro-Wilk test, indicated that the distribution of age and quantity of Anti-HCMV IgG was not normal (*p* < 0.001); therefore, the Mann-Whitney test was used to evaluate these variables. To compare the categorical variables of the study with Anti-HCMV IgG positivity, the Pearson Chi-Square test was employed. The correlation between age and the quantity of Anti-HCMV IgG was assessed using Spearman's Correlation. A significance level of 5 % was adopted.

## Results

The average age of the donors was 38.53 ± 12.17 years, with the majority being male (528/980, 53.9 %); 75 % (735/980) of the donors were from the city of Rio de Janeiro/RJ. Most had completed high school (369/980, 37.7 %) and were classified as blood type O+ (430/980, 43.9 %). The average CMV IgG level was 86.44 ± 45.95 U/mL, with 88.5 % (867/980) of the sample testing positive for this parameter (Table 1). All samples were negative for IgM, and HCMV DNA was detected in 1.2 % of the sample. All donors with detected HCMV DNA, except for one, were IgG-positive.

**Table 1:** Sociodemographic and laboratory characteristics of the donors.

Variables	n	%
Gender		
Male	528	53.9
Female	452	46.1
Age group		
18‒20	37	3.8
21‒30	260	26.5
31‒40	247	25.2
41‒50	265	27.0
51‒60	126	12.9
> 60	45	4.6
Education level		
Unlettered	2	0.2
Incomplete first grade	47	4.8
Complete first degree	48	4.9
Incomplete high school	48	4.9
Complete high school	369	37.7
Incomplete graduation	146	14.8
Complete graduation	320	32.7
BloodType		
O+	430	43.9
O-	55	5.6
A+	329	33,5
A-	33	3.4
B+	87	8.9
B-	16	1.6
AB+	27	2.8
AB-	3	0.3
IgG anti-HCMV		
Positive	867	88.5
Negative	113	11.5
HCMV DNA		
Positive	12	1.2
Negative	968	98.8
Total	980	100

Table 2 presents the association between the variables of gender, age, education level, and blood type with seropositivity for anti-HCMV IgG. We found no significant difference in positivity between genders. We observed that older patients were more positive for HCMV (IgG), a statistically significant result (*p* = 0.002). Regarding the ABO/Rh blood groups, the highest levels of positivity were observed in A- (97.0 %) and O+ (90.2 %) donors, while the lowest was in AB- donors (66.7 %). Donors who had started but not completed college showed a lower percentage of positivity (82.9 %) for anti-HCMV IgG compared to those who had not completed high school (93.4 %) (*p* < 0.001).

**Table 2:** Association between anti-HCMV IgG serological test results and gender, education level, and blood type.

Variables	IgG Anti-HCMV		Total	p^a^
	Positive, n (%)	Negative, n (%)		
Gender				
Male	464 (87.9)	64 (12.1)	528 (100)	0.532
Female	403 (89.2)	49 (10.8)	452 (100)	
Age				
≤25	142 (80.7)	34 (19.3)	176 (100)	0.002^b^
26‒60	684 (90.1)	75 (9.9)	759 (100)	
≥61	41 (91.1)	4 (8.9)	45 (100)	
School level				
Until high school	485 (93.4)	34 (6.6)	519 (100)	<0.001^b^
Graduation	382 (82.9)	79 (17.1)	461 (100)	
Blood type				
O+	388 (90,2)	42 (9,8)	430 (100)	0.130
O-	47 (85,5)	8 (14,5)	55 (100)	
A+	290 (88,1)	39 (11,9)	329 (100)	
A-	32 (97)	1 (3)	33 (100)	
B+	72 (82,8)	15 (17,2)	87 (100)	
B-	12 (75)	4 (25)	16 (100)	
AB+	24 (88,9)	3 (11,1)	27 (100)	
AB-	2 (66,7)	1 (33,3)	3 (100)	

a Pearson Chi-Square.

b Statistical significance.

Older patients exhibited higher positivity rates for anti-HCMV IgG. There was no correlation between age and HCMV DNA positivity. The levels of anti-HCMV IgG (U/mL) were higher in females and in patients with positive HCMV DNA, with statistically significant differences observed for gender (Table 3).

**Table 3:** Relationship of age to Anti-HCMV IgG and HCMV DNA results.

Variables	N	Mean ± SD	Rank average	p^a^
		Age		
HCMV DNA				
Positive	12	38.58 ± 14.20	486.04	0.956
Negative	968	38.52 ± 12.15	490.56	
				
IgG anti-HCMV				
Positive	867	38.94 ± 12.09	500.53	0.002^b^
Negative	113	35.32 ± 12.39	413.52	
Quantitative IgG anti-HCMV (U/mL)				
Gender				
Male	528	81.54 ± 44.18	457.09	<0.001^b^
Female	452	92.16 ± 47.34	529.53	
HCMV DNA				
Positive	12	88.46 ± 47.60	507.04	0.838
Negative	968	86.42 ± 45.95	490.29	

SD, Standard Deviation.

a Mann-Whitney test.

b Statistical significance.

Table 4 shows the serological status and viral load of donors in whom HCMV DNA was detected. The highest viral load was found in donors younger than 25 years old.

**Table 4:** Characterization of blood donors in whom HCMV DNA was detected.

Age	Gender	IgG (U/mL)	IgM (U/mL)	Viral load (copies mL)	CT
19	F	156	5.15	5.58E + 09	17.01
20	M	60.3	10	3.45E + 10	14.4
23	M	88.6	5.18	1.33E + 09	19.069
25	M	<5.00^a^	6.72	2.89E + 12	8.06
36	M	83.6	6,04	1.58E + 08	22.12
37	F	129	11.3	1.54E + 08	22.15
39	M	92.4	14.6	9.24E + 07	22.89
43	M	111	8.12	1.29E + 07	25.70
50	F	115	6.18	3.22E + 07	24.40
53	F	146	6.37	2.32E + 04	34.76
55	M	19.6	9.82	2.82E + 06	27.89
61	M	60.1	5.29	9.49E + 05	29.45

a Negative.

Although IgG titers tend to decline with age, the Spearman correlation between age and Anti-HCMV IgG titer was positive, albeit weak (Spearman correlation coefficient = 0.151; *p* < 0.001) (Table 4).

## Discussion

The prevalence of anti-HCMV IgG among blood donors was 88.5 %. This data is crucial for public health, enabling the development of strategies to prevent and control HCMV infections. Globally, the prevalence of anti-HCMV IgG is also high, especially in Asia, Africa, and South America, with rates of 82.64 %, 82.75 %, and 99.23 %, respectively.^21^^,^^22^ A study conducted in the Madinah region of Saudi Arabia found that 95.73 % of participants had anti-HCMV IgG antibodies.^8^ A meta-analysis reviewing global seroprevalence among blood donors indicated an overall rate of 83.16 %^21^ closely matching another systematic review that reported an 83 % global prevalence in the general population.^22^ A Canadian cross-sectional study showed a 70 % seroprevalence in blood donors over 70 years old, with an overall prevalence of 30 % among blood donors from Eastern Canada.^23^ In Australia, the prevalence among blood donors is 76.1 %, with older age groups exhibiting the highest rates.^24^

In 2009, Serra and collaborators reported a high prevalence of anti-HCMV IgG, 84 %, in 4620 samples from pregnant women in Brazil.^16^ TT-HCMV poses significant risks for immunocompromised patients, particularly after solid organ or bone marrow transplantation or during fetal life. The prevalence of anti-HCMV IgG among blood donors correlates with infection rates in the general population and the socioeconomic characteristics of donors. High seroprevalence indicates widespread past exposure to HCMV. Lower socioeconomic levels are associated with increased exposure to HCMV, linked to factors such as large families, small living spaces that promote crowding, inadequate childcare practices, and sexual behaviors.^25^^,^^26^

In our study, a higher level of education correlated with lower detection rates of IgG antibodies against HCMV (*p* < 0.001). This observation may be explained by the association between education level and access to information about preventive measures and proper hygiene to avoid HCMV infection. Individuals with higher education levels are likely to have better knowledge of infection risks and may adopt preventive behaviors such as regular hand washing, avoiding the sharing of contaminated utensils, and maintaining good personal hygiene.^26^ These practices can reduce exposure to the virus and, consequently, the likelihood of seroconversion and detection of IgG antibodies. Additionally, higher education levels may also correlate with easier access to health services and serological testing, potentially leading to increased detection of asymptomatic or subclinical infections, including IgG antibodies against HCMV.^26^

Our data also indicate a relationship between age and the presence of IgG antibodies against HCMV. The finding that older age groups are more likely to test positive for IgG suggests that the probability of exposure to the virus increases over a lifetime, in this study older patients were more positive for HCMV (IgG), a statistically significant result (*p* = 0.002). This is supported by the 2022 Mahallawi study, which found that the population displaying IgG antibodies against HCMV was predominantly aged between 30 and 61 years.^8^ The average age of participants in our study was 38.53 ± 12.17 years, indicating that our sample is primarily composed of middle-aged adults. It is well established that HCMV infection is common in the general population and that its prevalence increases with age. This is due to the virus's ability to persist in the body after the initial infection and to reactivate during periods of immunosuppression.^5^^,^^8^^,^^27^

Age is a significant risk factor for HCMV infection, particularly in older age groups, such as the elderly and immunocompromised individuals.^4^ In younger people, primary HCMV infection is more common, whereas older and immunocompromised adults are more likely to experience reactivation of the infection. The relationship found in our study between age and the presence of IgG antibodies against HCMV aligns with the general understanding of the virus's epidemiology, as previously described by several authors.^5^^,^^28^^,^^29^

We did not find any cases positive for anti-HCMV IgM, which contrasts with most previous studies on HCMV seroprevalence in blood donors. For instance, a study from Santa Catarina state in southern Brazil reported a 96.4 % IgG seroprevalence and 2.3 % IgM seroprevalence among blood donors.^14^

The absence of IgM-positive cases in our study could suggest that the infection was acquired sometime before the index blood donation or possibly indicates a reactivation, as most donors positive for HCMV DNA were also IgG-positive.

Molecular analysis data revealed that 12 (1.2 %) of the 980 samples tested were positive for HCMV DNA. Additionally, all samples tested for IgM were negative. Notably, the blood donors with the highest viral loads were negative for both anti-HCMV IgM and IgG. These findings highlight that asymptomatic blood donors can carry and transmit HCMV through their blood components. Donations made during the immunological window phase are believed to be the primary source of residual TT-HCMV infections in the post-leukocyte depletion era.^4^

Nucleic Acid Testing (NAT) for detecting HCMV in blood donors is an extremely useful tool for identifying HCMV viremia in blood donations. NAT is a sensitive and specific method that allows for the direct detection of HCMV genetic material in donor blood. It offers a more advanced screening approach compared to serological tests, such as detecting IgM or IgG antibodies, which may indicate past or current infection but do not confirm the direct presence of the virus.^3^^,^^11^^,^^30^

Considering that there are approximately 100,000 blood donations per year at HEMORIO, and on average, 1.5 cellular blood components (non-cellular blood components pose a very low or even nonexistent risk for HCMV transmission) are prepared from each donation, there is a theoretical risk of identifying 1500 positive blood components per year at this center (1.2 % positivity rate × 150,000 blood component units). These 1500 blood components could potentially transmit HCMV to transfusion recipients, posing a significant risk of irreversible damage and even death for high-risk patients.

This rate is notably high compared to published data from other countries. In the USA, a study by Bush et al. reported a rate of 0 2 %; in Germany, the rate of HCMV viremia is even lower at 0.09 %, while in China, a metagenomic analysis of samples from 5.734 blood donors identified a rate of 0.76 %.^17^^,^^31^^,^^32^

Brazilian guidelines stipulate that blood components intended for use in newborns and intrauterine transfusions, individuals who have undergone hematopoietic stem cell or organ transplants, and newborns of HCMV-negative mothers or with negative or unknown HCMV serology results who weigh less than 1200 grams should only receive CMV-negative or leukodepleted blood. Moreover, the ordinance allows leukocyte-depleted cellular components to replace the use of CMV-negative components. Additionally, CMV serology results must be indicated on the labels of the blood component bags. However, Brazilian guidelines do not address the use for other patient groups who are also at risk for symptomatic and severe HCMV infections, such as individuals with AIDS.

According to our findings, serology may not be the most effective method to prevent CMV transmission through transfusions, as we identified that 1.2 % of donors tested positive for HCMV DNA but negative for anti-HCMV IgG. Universal NAT or universal leukodepletion would be more effective strategies to mitigate this potentially serious infectious risk from blood transfusions.

The gold standard for diagnosing HCMV is the Quantitative Nucleic Acid Test (QNAT). QNAT-HCMV is typically performed using Real-Time PCR (RT-PCR) on plasma or whole blood. While detecting HCMV DNA in donor blood would increase the safety of blood transfusions, the prevalence of HCMV IgG in the general population should be considered before implementing such a test ‒ and no country has yet made HCMV NAT mandatory for blood donors.

Universal leukoreduction appears to be a more feasible measure since it can prevent not only HCMV transmission but also other infections, such as those caused by Leishmania and potentially HTLV and *Trypanosoma cruzi*. In light of this, and because leukodepletion offers additional benefits like preventing Febrile Non–Hemolytic Transfusion Reactions and HLA alloimmunization, many countries mandated this method as a standard practice many years ago.

It is important to note that these projections are based on provided information and assumptions, such as the number of bags of blood components generated per donor. Factors such as screening criteria and the distribution of bags may also influence the actual number of patients receiving these bags and the disposal of samples. These projections should be viewed as estimates based on available data. It is also important to emphasize that HCMV is a virus that infects individuals of all ages, typically causing primary infections that are generally asymptomatic. After primary infection, the virus becomes latent within the body, and episodes of reactivation can occur during periods of immunosuppression.

In conclusion, the number of DNA-positive samples was notably high at 1.2 %, which is significantly higher than the prevalence of any other infectious agent routinely tested for in the Brazilian blood system through NAT, including HIV, HCV, HBV, and *Plasmodium spp*. Moreover, the high seroprevalence underscores the risk of HCMV transfusion-transmission in Brazil because even non-viremic but seropositive donors could theoretically transmit HCMV or trigger virus reactivation in recipients. These findings emphasize the importance of detecting infections and the potential to transmit the virus to susceptible individuals. They also highlight the need for ongoing surveillance, prevention, and strategies to minimize and screen for HCMV transmission through blood transfusions.

## Funding

Coordenação de Aperfeiçoamento de Pessoal de Nível Superior ‒ Brasil (CAPES) – Finance Code 001, Fundação Carlos Chagas Filho de Amparo à Pesquisa do Estado do Rio de Janeiro (FAPERJ), Conselho Nacional de Desenvolvimento Científico e Tecnológico (CNPq), Instituto Oswaldo Cruz and Instituto de Hematologia Arthur de Siqueira Cavalcanti/HEMORIO.

## Conflicts of interest

The authors declare no conflicts of interest.
